# Kurdish standard EMNIST-like character dataset

**DOI:** 10.1016/j.dib.2024.110038

**Published:** 2024-01-09

**Authors:** Hamsa D. Majeed, Goran Saman Nariman, Renas Sardar Azeez, Bawar Bilal Abdulqadir

**Affiliations:** Department of Information Technology, College of Science and Technology, University of Human Development, Kurdistan Region, Iraq

**Keywords:** Kurdish characters, Handwritten character recognition, Central Kurdish, Optical character recognition, Kurdish handwritten

## Abstract

A dataset was created by collecting handwritten samples of distinct Kurdish characters. The dataset consists primarily of 58 characters, and approximately 3800 adult volunteers who are native Kurdish speakers participated in the collection process. Each participant was requested to fill two rows in a character form printed on A4 landscape papers. These papers were divided into sets of four pages, with 18 columns and 10 rows of characters on each page, except for the fourth page in each set, which had 40 cells. To ensure a comprehensive dataset, over 760 sets were prepared and distributed across various universities and institutions. The collected samples underwent scanning, cropping, and preprocessing procedures following the characteristics established by the EMNIST project. The purpose of these procedures was to standardize the dataset and ensure uniformity in the representation of all characters.

Specifications Table

**Table:** 

Subject	*Artificial Intelligence*
Specific subject area	Offline Handwritten Optical Character Recognition
Data format	Analysed, Filtered
Type of data	Image
Data collection	A printed grid form of A4 papers was prepared that each character was written on, then filled by volunteers (each volunteer filled out only two rows by pen). Following this, they were scanned by a scanner with the same quality, and each character was accurately segmented. Finally, the characters were organized into separate corresponding folders, preserving the exact size of each character file.
Data source location	Sulaymaniyah, Kurdistan Region, Iraq.
Data accessibility	Kurdish Standard Characters EMNIST-like Dataset [1]. Data identification number: 10.17632/d2j939k88t.1 Direct URL to data: https://data.mendeley.com/datasets/d2j939k88t/1

## 1 Value of the Data

• This dataset can facilitate interdisciplinary research, bringing together experts in linguistics, computer science, cultural studies, and education to explore various aspects of the Kurdish language and script.
• The EMNIST-like characteristics of this dataset make it poised to serve as a benchmark, particularly in the domain of machine learning models focused on handwritten character recognition. This Kurdish character dataset is positioned to play a crucial role in assessing and enhancing the performance of machine learning algorithms in the context of Kurdish script recognition.
• The existence of all the variations and configurations for each letter within the Kurdish characters in this dataset, filled the gap in the existing Kurdish standard dataset for the fields of handwritten recognition and identification.
• This dataset holds significant relevance within the realm of technology aimed at converting handwritten Kurdish script into audible speech, making it a valuable resource for advancing the development of applications in this specific field. Moreover, it serves as a pivotal building block for creating effective and accurate handwritten-to-voice conversion systems tailored to the Kurdish script.
• Due to its comprehensive nature, this dataset serves as an excellent foundational resource when delving into word recognition. Its exhaustive coverage of all possible character shapes within the Kurdish script makes it an ideal reference point for researchers seeking to explore and address the multifaceted challenges associated with recognizing words in Kurdish script.
• Introducing a fresh research area for machine learning algorithms involves the use of different language script datasets, characterized by equivalent quantity and quality attributes. A notable example of such research entails investigating the performance of machine learnability across various scripts.

## 2 Data Description

The Kurdish language has two writing systems: Latin and Arabic alphabets. The Arabic scripts are used in Iraq and Iran, and the Latin scripts are used in Syria, Armenia, and Turkey. This dataset is restricted to Sorani (Central Kurdish) which is the standard used dialect for writing the Kurdish language from past to present. It is spoken by an estimated 9 to 10 million people in both Iran and Iraq. Furthermore, one intriguing aspect of Sorani Kurdish script is its cursive nature, which implies that some characters may not have the same shapes depending on where they are positioned inside a word [2,3]. However, the dataset has addressed this concern by encompassing a comprehensive array of character shapes, ensuring that it accounts for the full spectrum of variations encountered in Sorani cursive writing.

The dataset mainly consists of (58) characters with the help of circa (3800) native individuals. A vast collection of isolated handwritten Central Kurdish character images has been assembled in this work. It has (440800) images, with (7600) images for each character. To protect the dataset's authenticity and quality, no augmentation was used to make it bigger.

Over the past two decades, the handwriting recognition community has created numerous benchmark handwritten character databases to facilitate research, allowing for the development, evaluation, and comparison of different recognition methods. A comprehensive survey of these handwriting databases, presented by [4], offers insights into their statistics, characteristics, and supported tasks. It also provides a comparative analysis of these databases across various dimensions. Additionally, it is worth mentioning datasets presented in recent literature that the researchers utilized for the purpose of handwritten recognition, like [5] and [6], as the primary objective of this work.

The more difficult classification challenge represented by Extended MNIST (EMNIST), which employs the same conversion paradigm as the MNIST dataset, has become a benchmark for learning, classification, and computer vision systems [7]. The dataset represented in this work is pre-processed and then prepared following EMNIST characteristics. The primary point to consider for the collecting procedure was the NIST dataset, which included at least 3600 writers [8]. Table 1 displays all the characters involved in the dataset and the number of images for each character.

**Table 1:** List of the characters, the name of a folder with the name of the letter in it, and the total number of images for each letter.

A zip file containing the whole dataset that is described in this paper besides individual folders for each character along with separated folder names (Samples) created in the Mendeley Repository.

Manual refinement has been applied to the dataset to overcome the Instances of miswriting, incorrect entries, excessive dirt, blank cells, excessively small writing, missing parts, missing dots, etc. that were discovered within the collected forms. Yield into a varying number of characters as Table 1 illustrates with a minimum of 7000 samples of the individual characters. Consequently, a considerable amount of effort was spent manually removing any unsuitable data from each character set.

Other datasets for Kurdish characters exist but they only include standalone characters, omitting various possible shapes and covering only the main 35 characters. In contrast, the dataset presented in this work covers all possible shapes of each character, regardless of their position within a word. Furthermore, the creation of this database adheres to EMNIST characteristics with additional preprocessing steps that have been incorporated and will be discussed in the upcoming section.

It is worth mentioning that a significant similarity is observed between the characters ``'' and ``ك''. Considering that a handwriting dataset aims to enhance the accuracy of a recognition model, it is reasonable to exclude the character "**ك**" (folder named ``K'' in Table 1) during the training of a recognition model. This decision is based on two facts:

1. There is no such word in Kurdish that ends with  therefore when detecting it in the end it can be classified as ك.
2. In the new Kurdish written system, the ك has been removed and has not been in use anymore, only ک is in use.

## 3 Experimental Design, Materials and Methods

### 3.1 Collecting data

**Fig. 1:**
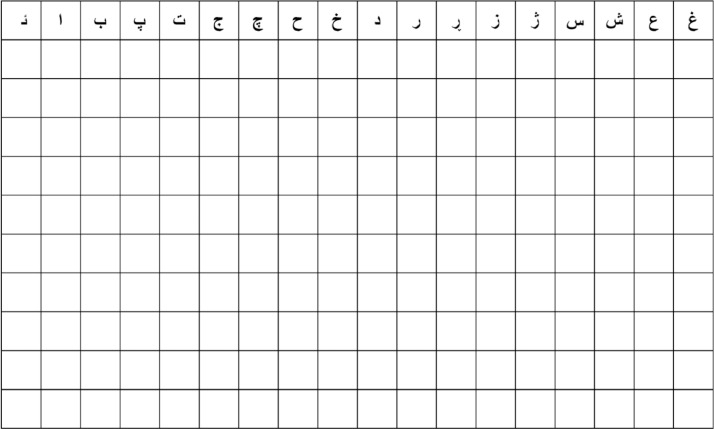
Sample of prepared characters form.

The dataset has been collected with the help of around (3800) native individuals asked to fill two rows respectively in the prepared characters` form presented in Fig. 1, in other words, from each participant, two samples of each character have been collected. The individuals filled one row at a time covering all the characters, sequentially back to the first character in the second row assigned to that participant to be filled with the aim of avoiding similarity between the consecutive rows. Furthermore, there are secondary instructions given during the data collection like keeping the form clean, being written in the usual manner without being slowed down, or emphasizing the creation of visually pleasing letter forms, each character is written in one cell within its border without crossing to the adjacent cell as possible. A total of around (440800) images were collected.

The primary value of this work lies in the method of collection. As previously mentioned, only two samples were required from each volunteer. This approach ensures that the same writer’s handwriting does not appear in both the training and testing phases when creating a machine learning model. By limiting the number of samples per person to two, the trained model becomes more reliable and valid, as the likelihood of encountering the same writer in both the training and testing phases is significantly reduced. In contrast, if ten samples were taken from each individual (which makes the collection process much easier) the probability of the same writer appearing in both phases would be unacceptably high, leading to an inaccurate and invalid model. Additionally, finding a sufficient number of volunteers to complete the form ten times would be difficult.

The characters grid form is prepared on A4 landscape papers in which 18 columns of characters with 10 rows in each paper are grouped into a set of 4 pages covering all 58 characters with 180 cells in each paper to be filled except for the fourth page with 40 cells. In the end, each set provides 580 samples, 10 samples for each character. In order to collect at least 7000 samples for each character. 760 sets were prepared and distributed over many universities and institutions as presented in Table 2. The age of participants varied between 18 and 25 years, encompassing individuals of both male and female genders.

**Table 2:** Source of data.

University/ Institution	Place (City/Country)
University of Human Development	Sulaymaniyah / Iraq
University Of Sulaimani	Sulaymaniyah / Iraq
Qaiwan International University/UTM	Sulaymaniyah / Iraq
Blnd High School	Sulaymaniyah / Iraq
Zanst High School	Sulaymaniyah / Iraq

**Fig. 2:**
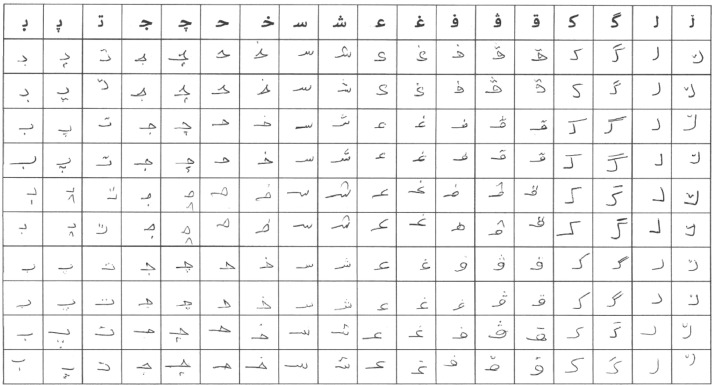
Sample of scanned characters form.

All characters` grid pages were scanned using a high-quality scanner, the output scanned image was saved in jpg format because the capability of compression makes it more convenient for the storage matter. All the letters were written in a black or dark blue pen since the paper was white. An example of a scanned form is shown in Fig. 2.

The forms were scanned using high-definition mode, resulting in the creation of four folders, each containing approximately 750 images of a single form page. The scanning process required careful execution to prevent any vertical or horizontal obliqueness.

Following the scanning phase, a cropping process was implemented for each character grid page to isolate individual letter blocks. This involved the development of a simple Java program designed to crop each cell within the grid. The program systematically accessed each folder, processing one image at a time to crop all handwritten cells, excluding the first cell, which contained the keyboard-printed character that already existed in the form. The cropping process occurred row by row, and each row was saved in a designated folder with corresponding character labels. The entire letter images were saved in the same size. The size of the scanned page forms is (3456*2448) pixels, while the cropped cell sizes (letters) are (182*160) pixels.

### 3.2 Preprocessing data

The pre-processing phase is important in any recognition system. The goal of the preprocessing process is to improve the quality of the images for extracting the proper features later in any recognition system. A preprocessing process was applied to each cropped letter to enhance the images.

**Fig. 3:**
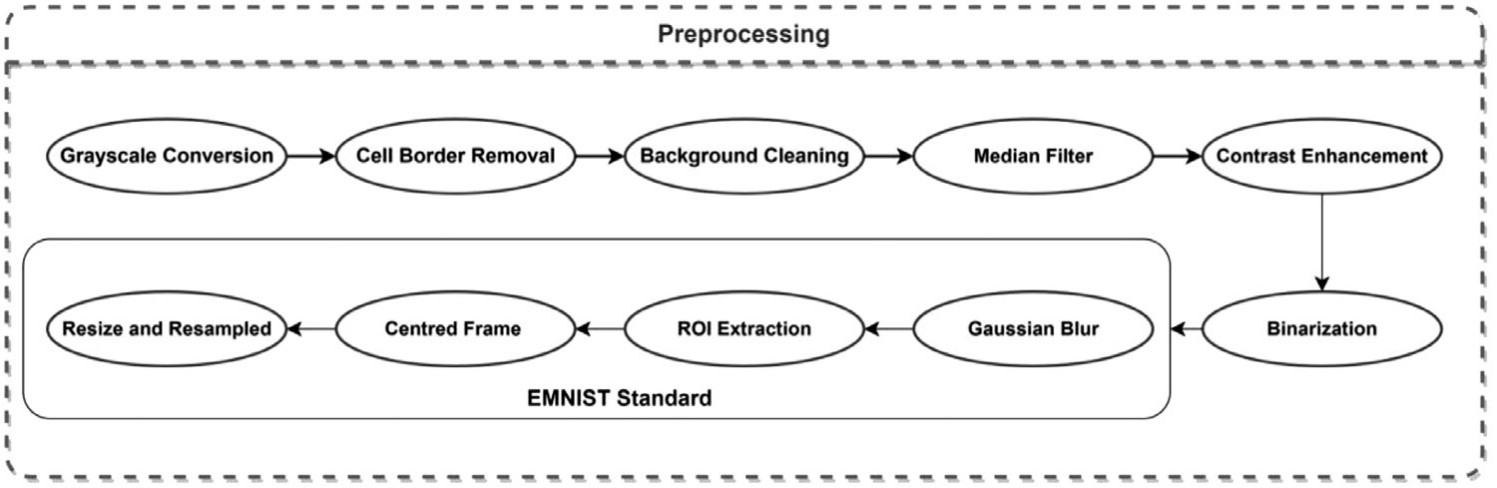
Block diagram of the preprocessing steps.

The collected handwritten dataset is challenging and needs proper preprocessing techniques to ensure the optimum result which is a high-level clear and clean image. The preprocessing steps involved in this work are illustrated in Fig. 3.

#### 3.2.1 Grayscale conversion

It is a digital photography technique applied to character image conversion. All color information is removed, leaving only various shades of gray, with white being the lightest and black being the darkest. The brightness of the image is represented by each pixel.

#### 3.2.2 Median filter

The median filter is used as a noise reduction procedure to eliminate noise from character images and enhance subsequent processing outcomes.

#### 3.2.3 Contrast enhancement

Contrast enhancements increase the visibility of objects in the character image, commonly conducted as a contrast stretch followed by a tonal enhancement. Boosts the brightness differences on one hand and improves the brightness differences equally across the dynamic range of the image on the other hand.

#### 3.2.4 Binarization

Character image is converted into a two-tone image using the binarization method. The process is performed based on a global-single threshold with a mean value equal to 220. Additionally, another step applied to the aim of reconstruction of the broken pixels if any.

#### 3.2.5 EMNIST characteristics

**Fig. 4:**
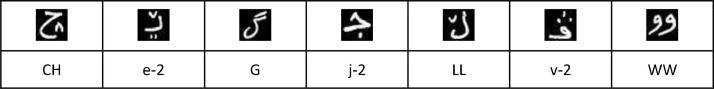
Samples of the outcome with their labels.

Starting from this point the procedure of preprocessing followed the EMNIST characteristics [7], as shown in Fig. 3. Starting with Gaussian blur which is applied to reduce noise and smooth out fine details. Then the character in the image is fitted with a bounding box (ROI) and then extracted. The aspect ratio of the extracted region of interest is kept, and it is centred within a square frame with lengths equal to the larger dimension. The image is then downscaled to 24 × 24 pixels. Then a 2-pixel empty padding is applied to this square frame, two pixels for each side, resulting in 28 × 28 pixels images as Fig. 4 shows.

## Limitations

Collecting a massive handwritten character dataset requires addressing limitations related to availability, and variations in handwriting styles.

The availability of handwritten Kurdish characters may be limited compared to more widely spoken languages. Kurdish is primarily spoken in certain regions. This scarcity of resources can pose challenges in acquiring a diverse and comprehensive dataset.

Variations in Kurdish handwriting styles can present difficulties in ensuring the dataset's quality and consistency. It poses several challenges due to the inherent complexity and variability of human handwriting. It exhibits a wide range of styles, shapes, and variations among individuals. Each person has a unique way of forming characters, resulting in significant variability even for the same letter. Consequently, a manual refinement process becomes necessary to address cases of miswriting, incorrect entries, excessively small writing, missing parts and/or dots, and other identified issues found within the collected characters.

## Ethics Statement

This work submitted according to the institutional guidelines of the University of Human Development. The study does not involve human subjects, animal experiments, or any data collected from social media platforms. All handwriting samples have been collected with the consent of the individuals and their respective institutions. These individuals participated voluntarily, and no interventions were conducted during the writing process.

## CRediT Author Statement

**Hamsa D. Majeed:** Data processing, Writing, Validation, Supervision, Review, Refinement, and Editing.

**Goran Saman Nariman:** Conception, Writing, Validation, Supervision, Refinement, Review, and Editing.

**Renas Sardar Aziz:** Data collecting, refinement, and management.

**Bawar Bilal Abdulqadir:** Data collecting and management

## Data Availability

Kurdish Standard EMNIST-like Character Dataset (Original data) (Mendeley Data)
